# Characterizing Thalamocortical Disturbances in Cervical Spondylotic Myelopathy: Revealed by Functional Connectivity under Two Slow Frequency Bands

**DOI:** 10.1371/journal.pone.0125913

**Published:** 2015-06-08

**Authors:** Fuqing Zhou, Lin Wu, Xiaojia Liu, Honghan Gong, Keith Dip-Kei Luk, Yong Hu

**Affiliations:** 1 Department of Radiology, the First Affiliated Hospital, NanChang University, Nanchang, Jiangxi, China; 2 Department of Orthopaedics and Traumatology, Li Ka Shing Faculty of Medicine, The University of Hong Kong, Pokfulam, Hong Kong; UCLA, UNITED STATES

## Abstract

**Background and Purpose:**

Recent advanced MRI studies on cervical spondylotic myelopathy (CSM) revealed alterations of sensorimotor cortex, but the disturbances of large-scale thalamocortical systems remains elusive. The purpose of this study was to characterizing the CSM-related thalamocortical disturbances, which were associated with spinal cord structural injury, and clinical measures.

**Methods:**

A total of 17 patients with degenerative CSM and well-matched control subjects participated. Thalamocortical disturbances were quantified using thalamus seed-based functional connectivity in two distinct low frequencies bands (slow-5 and slow-4), with different neural manifestations. The clinical measures were evaluated by Japanese Orthopaedic Association (JOA) score system and Neck Disability Index (NDI) questionnaires.

**Results:**

Decreased functional connectivity was found in the thalamo-motor, -somatosensory, and -temporal circuits in the slow-5 band, indicating impairment of thalamo-cortical circuit degeneration or axon/synaptic impairment. By contrast, increased functional connectivity between thalami and the bilateral primary motor (M1), primary and secondary somatosensory (S1/S2), premotor cortex (PMC), and right temporal cortex was detected in the slow-4 band, and were associated with higher fractional anisotropy values in the cervical cord, corresponding to mild spinal cord structural injury.

**Conclusions:**

These thalamocortical disturbances revealed by two slow frequency bands inform basic understanding and vital clues about the sensorimotor dysfunction in CSM. Further work is needed to evaluate its contribution in central functional reorganization during spinal cord degeneration.

## Introduction

Cervical spondylosis myelopathy (CSM) is the most common disorder with chronic spinal cord compression in older people. It is associated with weakness or sensory loss in one or more limbs, even pain [1]. Limited understanding of neuroplasticity of CSM has constrained development of rehabilitative treatments because it is usually considered as a disease with local compressive impairment, neglecting the intimate interconnection with the cerebral cortex.

In patients with CSM, some studies have confirmed increased activation in the primary motor cortex [2–4] or activation loss in the sensory cortex[3], accompanied with an increased amplitude of cortical low-frequency oscillations[5] and decreased neuronal metabolite (N-acetylaspartate/creatine ratio)[6]. Growing evidence implicates significant thalamocortical communication disturbances in spinal cord injury (SCI)[7,8]. Indeed, CSM is partly regarded as a specific incomplete SCI. However, the thalamocortical functional connectivity in patients with CSM remains unclear.

Current in resting-state functional magnetic resonance imaging (rs-fMRI) studies, functional connectivity is defined as temporal correlation between physically distant brainregions, and it has always been a concern that functional connectivity is mostly confined to frequencies<0.1Hzcontribute to correlated activity [9]. According to Buzsáki framework [10], which allows us differentiate rs-fMRI signal into four frequency bands: slow 2–5. By contrast with the physiological fMRI signal in low frequencies (including slow-5 and slow-4), the contributions of signal in high (>0.1Hz, including slow 2–3) frequencies are minor to functional connectivity [9]. More interestingly, several studies have investigated the distinct frequency-specific characteristics of functional connectivity in regional [11], interregional [12], and network levels[13] of different brain areas. The neurophysiological mechanism underlying distinct frequency-specific connectivity properties may arise from the assorted cytoarchitecture or axon/synaptic types in these areas, linking specific neural processes, including input selection, synaptic plasticity, and long-term consolidation of information[11,14,15], but the exact mechanism remain poorly understood. The neural manifestations of connectivity property are distinct between slow-4 (0.027–0.073 Hz) and slow-5 (0.01–0.027 Hz), which was also addressed in subcortical and sensorimotor regions [11–13].In this study, the thalamocortical communication in patients with CSM we investigated through resting-state functional connectivity (rsFC)including: (1) examine thalamic coupling via individual-specific anatomically derived thalamic seeds to obtain a comprehensive cortex-wide assay of disturbances. Based on previous reports [5,10], thalamocortical connectivity properties under slow-4 and slow-5 frequency band could inform basic understanding of neural dysfunction or plasticity in thalamocortical circuits. In this study, we hypothesized thalamocortical disturbances across sensory- and motor- circuits, could revealed by functional connectivity under two slow frequency bands. Match-well groups CSM patients and health control (n = 17) rs-fMRI data was preprocessing, time-series were subdivided by band-pass filtering into a relatively lower frequency slow-5 and a slightly higher frequency slow-4 band for functional connectivity analysis; (2) evaluation on whether observed thalamocortical disturbances scale with symptoms or the severity of spinal cord damage. This study could provide a new insight into understanding of the pathophysiology of thalamo-cortical circuits of CSM patients.

## Results

### Demographic and clinical data

The demographic and clinical data (Data A in S2 File) of the study groups are listed in Table 1. The groups were not different with respect to age (*P* = 0.99) or sex (*P* = 0.92). Patients presented loss of dexterity in the hands and gait dysfunction. CSM and control groups had significantly different Neck Disability Index (NDI) scores and Japanese Orthopaedic Association (JOA) scores (S1 File). Decreased JOA scores in patients (11.82±2.81) were corresponding to disabling cervical compressive myelopathy, while alteration of NDI in patients (32.6%±11.9%) also means the seriousness of neck-related syndrome.

**Table 1:** Demographic data and clinical measures scores for cervicalspondylotic myelopathy group and healthy controls.

*Subject*	*CSM*	*HC*	*P-value*
*n*	*17*	*17*	*n/a*
*Age*	*50*.*53±7*.*27*	*50*.*26±7*.*31*	*0*.*99*
*Gender (male/female)*	*9/8*	*8/9*	*0*.*92*
*Handedness (right/left)*	*17/0*	*17/0*	*n/a*
*Duration of symptoms (month)*	*9*.*06±9*.*86*	*n/a*	*n/a*
*JOA scores*	*11*.*82±2*.*81*	*17±0*	*<0*.*0001*
* Motor upper*	*2*.*17±0*.*81*	*4±0*	*<0*.*0001*
* Motor lower*	*3*.*35±1*.*17*	*4±0*	*<0*.*0001*
* Sensory deficit*	*2*.*94±0*.*24*	*6±0*	*<0*.*0001*
* Bladder dysfunction*	*3*.*35±0*.*86*	*3±0*	*<0*.*0001*
*NDI Score*	*32*.*6%±11*.*9%*	*1*.*3%±0*.*5%*	*<0*.*0001*
*FA values*			
* FA values in C2 level*	*0*.*595±0*.*039*	*0*.*662±0*.*042*	*0*.*016*
* FA values in the severe level*	*0*.*497±0*.*064*	*n/a*	*n/a*

n/a = not applicable; JOA = Japanese Orthopaedic Association; NDI = Neck Disability Index; FA = Fractional Anisotropy; C = Cervicalvertebra; CSM = cervical spondylotic myelopathy; HC = healthy controls.

### Thalamocortical dysconnectivity in the slow-5 band

**Fig 1:**
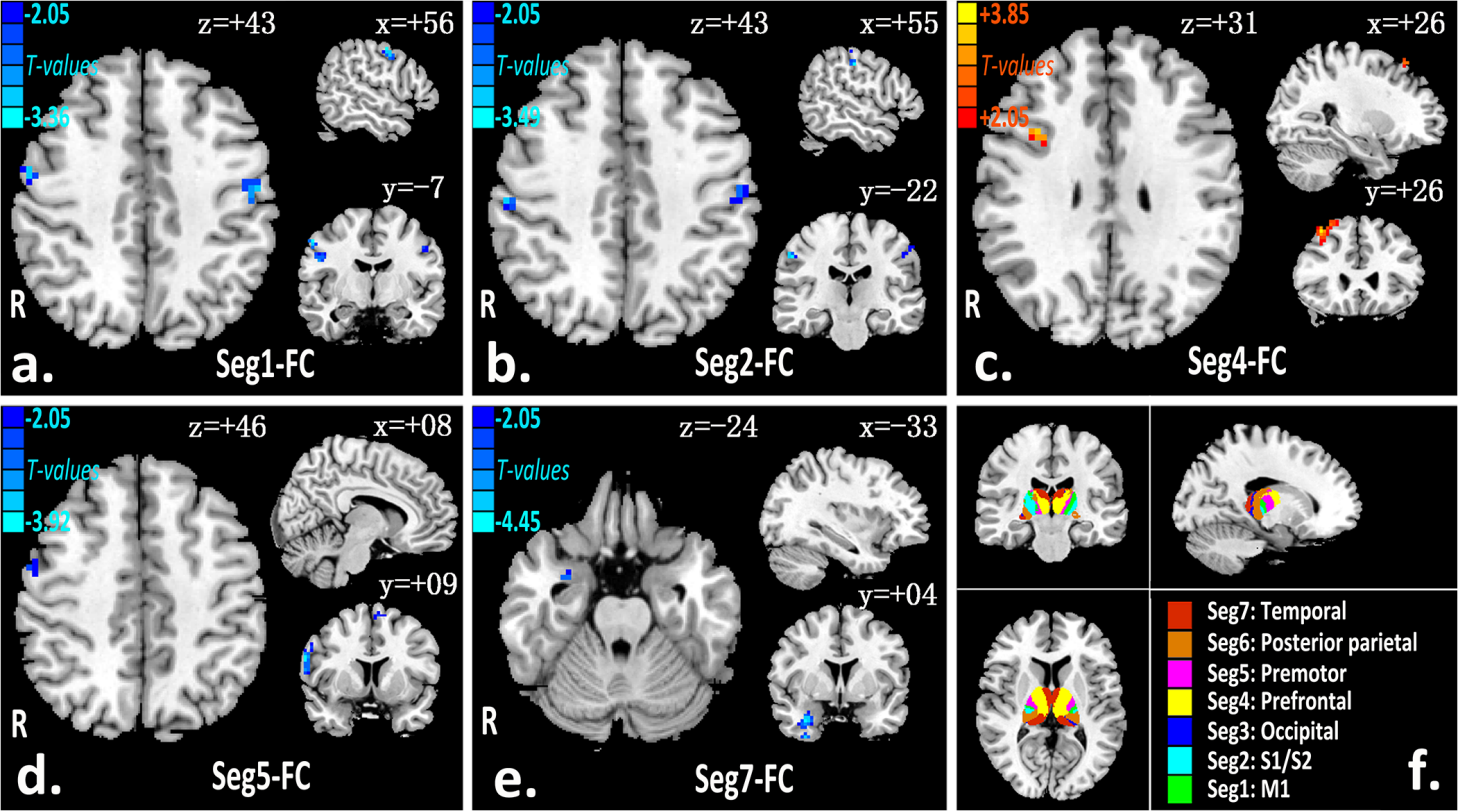
Thalamic dysconnectivity patterns in slow-5 frequency-band (a-e). There were significantly dysconnectivity between the thalamic segments (number 1, 2, 4, 5, 7, respective) and primary motor cortex (P < 0.05, AlphaSim corrected; cluster size > = 20), primary and secondary somatosensory (P < 0.05, AlphaSim corrected; cluster size > = 16), prefrontal (P < 0.05, AlphaSim corrected; cluster size > = 90), premotor (lateral and medial) (P < 0.05, AlphaSim corrected; cluster size > = 50), and temporal (P <0.05, AlphaSim corrected; cluster size > = 80). Functional connected seeding from bilateral anatomy connectivity-based thalamic segmentation templates (f) to the exclusive cortex.

Fig 1A–1E shows the robust between-group difference of rsFC between the thalamic subfields and its exclusive cortex in the slow-5 band. Compared with healthy controls, CSM patients with decreased rsFC between the thalamus and bilateral primary motor (M1) (Fig 1A), bilateral primary and secondary somatosensory (S1/S2) (Fig 1B), bilateral premotor cortex (PMC) (Fig 1D), or right temporal cortex (Fig 1E); but increased rsFC between the thalamus and right prefrontal cortex (PFC) (Fig 1C). No region with altered rsFC was found between the thalamic segments and the occipital or posterior parietal cortices in the CSM group. The differences of thalamocortical connectivity in t-values and cluster size of the patient vs healthy controls in the slow-5 band are listed in Table 2.

**Table 2:** The cervical spondylotic myelopathy patients compared with the controls, brain areas of thalamocortical dysconnectivity in the slow-5 frequency-band (p< 0.05, corrected with AlphaSim).

*The exclusive cortex*	*BA*	*Brain regions*	*Peak intensity-value*	*Number of voxels*	*Peak location (MNI)*		
					*x*	*y*	*z*
*CSM patients< Health controls*							
*Primary motor cortex*	*4*	*Left precentralgyrus*	*-3*.*22*	*37*	*-54*	*-15*	*45*
	*4*	*Right precentralgyrus*	*-3*.*36*	*36*	*57*	*-6*	*45*
*Primary and secondary somatosensory*	*3*	*Left postcentral gyrus*	*-2*.*70*	*18*	*-54*	*-18*	*42*
	*3*	*Right postcentral gyrus*	*-3*.*49*	*16*	*54*	*-21*	*39*
*Premotor*	*6*	*Right inferior frontal gyrus*	*-3*.*92*	*83*	*63*	*9*	*24*
	*6*	*Left superior frontal gyrus/ supplementary motor area*	*-3*.*16*	*81*	*-12*	*3*	*66*
*Temporal*	*21*,*38*	*Right middle temporal pole*	*-4*.*45*	*169*	*30*	*0*	*-33*
	*27*	*Right parahippocampagyrus*	*-4*.*36*	*132*	*15*	*-36*	*3*
*CSM patients > Health controls*							
*Prefrontal*	*8*,*9*	*Right middle frontal gyrus*	*3*.*85*	*123*	*36*	*27*	*51*

Notes: BA = Brodmann area; MNI = Montreal neurological institute; CSM = cervical spondylotic myelopathy

### Thalamocortical hyperconnectivity in the slow-4 band

**Fig 2:**
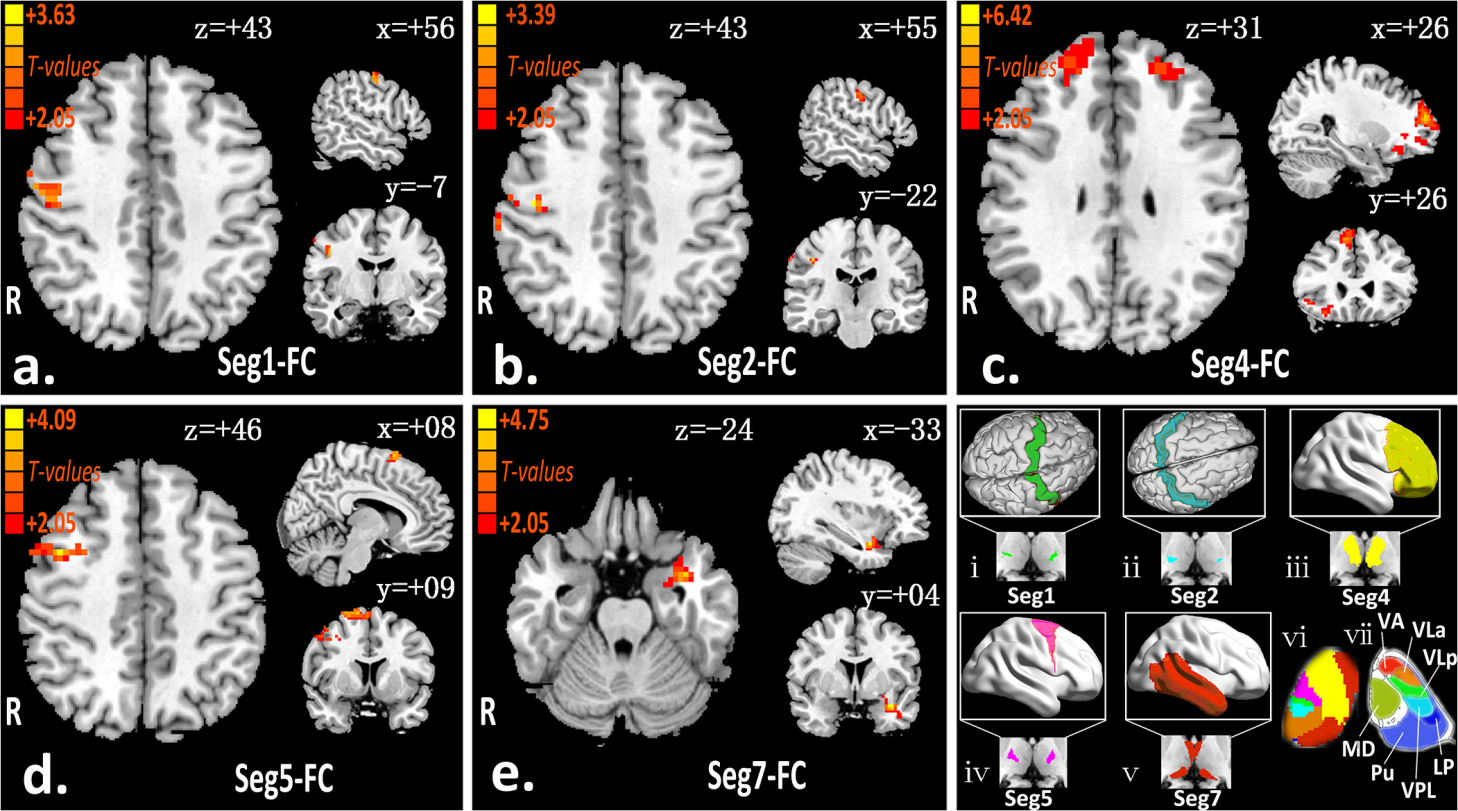
Thalamic hyperconnectivity patterns in slow-4 frequency-band (a-e). There were significantly hyperconnectivity between the thalamic segments (number 1, 2, 4, 5, 7, respective) and primary motor cortex (P < 0.05, AlphaSim corrected; cluster size > = 20), primary and secondary somatosensory (P < 0.05, AlphaSim corrected; cluster size > = 16), prefrontal (P < 0.05, AlphaSim corrected; cluster size > = 90), premotor (lateral and medial) (P < 0.05, AlphaSim corrected; cluster size > = 50), and temporal (P < 0.05, AlphaSim corrected; cluster size > = 80). Cortical subdivisions(ⅰ-ⅴ): green = M1 (corresponding to Seg1); cyan = S1/S2 (corresponding to Seg2); yellow = PFC (corresponding to Seg4); magenta = PMC (corresponding to Seg5); red = temporal (corresponding to Seg7); (ⅵ) Seven segmentation of the human thalamus base on anatomy connectivity. (ⅶ) An axial sample of thalamic section from the cytoarchitectonic atlas[16]. VA = ventral anterior nucleus; VLa = ventral lateral anterior nucleus; VLp = ventral lateral posterior nucleus; VPL = ventral posterior lateral nucleus; LP = lateral posterior nucleus; Pu = pulvinar; MD = mediodorsal nucleus.

**Fig 3:**
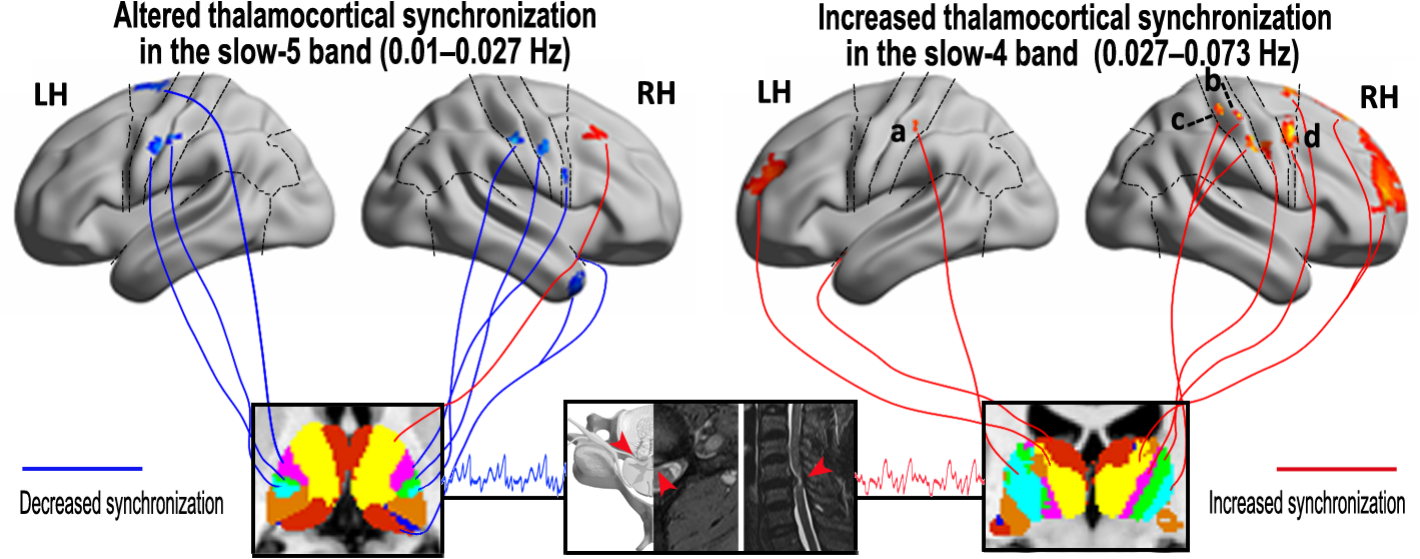
Exemplary surface maps of thalamocortical disturbances in two slow frequency bands. The difference of thalamo-cortical rsFC in slow-5 and slow-4 bands, between the patients with CSM versus controls, red and blue colors denote increased and decreased connectivity coefficients, respectively. (LH, left hemisphere; RH, right hemisphere).

Fig 2A–2E shows the robust between-group difference of rsFC between the thalamic subfields and its exclusive cortex in the slow-4 band. Compared with healthy controls, CSM patients showed increased rsFC between the thalamus and right M1 (Fig 2A), bilateral S1/S2 (Fig 2B), bilateral PFC (Fig 2C), right PMC (Fig 2D), and left temporal cortex (Fig 2E). No region with altered rsFC was found between the thalamic segments and the occipital, or posterior parietal cortices in the CSM group. The differences of thalamocortical connectivity in t-values and the cluster size of the patient vs healthy controls in the slow-4 band are listed in Table 3. Fig 3 summarize the surface-wise thalamocortical disturbances revealed by two slow frequency bands (slow-4 and slow-5) in patients with CSM.

**Table 3:** The cervical spondylotic myelopathy patients compared with the controls, brain areas of thalamocortical hyperconnectivity in the slow-4 frequency-band (p< 0.05, corrected with AlphaSim).

*The exclusive cortex*	*BA*	*Brain regions*	*Peak intensity-value*	*Number of voxels*	*Peak location (MNI)*		
					*x*	*y*	*z*
*CSM patients > Health Controls*							
*Primary motor cortex*	*4*	*Right precentralgyrus*	*3*.*63*	*72*	*48*	*-15*	*45*
*Primary and secondary somatosensory*	*3*	*Rightpostcentral gyrus-1*	*3*.*39*	*47*	*45*	*-15*	*42*
	*3*	*Rightpostcentral gyrus-2*	*3*.*16*	*25*	*66*	*-27*	*36*
	*3*	*Right postcentral gyrus-3*	*2*.*55*	*20*	*39*	*-39*	*66*
	*2*	*Left postcentral gyrus*	*3*.*04*	*16*	*-51*	*-30*	*51*
*Prefrontal*	*10*	*Right superior/middle frontal gyrus*	*5*.*55*	*448*	*30*	*57*	*15*
	*8*	*Right superior frontal gyrus*	*6*.*42*	*118*	*12*	*33*	*54*
	*10*	*Left superior frontal gyrus*	*4*.*12*	*114*	*-33*	*42*	*24*
*Premotor*	*6*	*Right superior frontal gyrus*	*4*.*03*	*81*	*21*	*12*	*66*
	*6*	*Right middle frontal gyrus*	*4*.*09*	*77*	*42*	*6*	*45*
*Temporal*	*38*	*Left superior temporal gyrus*	*4*.*75*	*80*	*-36*	*3*	*-21*

Notes: BA = Brodmann area; MNI = Montreal neurological institute; CSM = cervical spondylotic myelopathy

### Clinical associations of thalamocortical connectivity in CSM

In the slow-5 band, there were no correlations between the rsFC and the duration of symptoms (*P* = 0.144 to 0.911), JOA score (*P* = 0.247 to 0.974), NDI score (*P* = 0.190 to 0.981), the FA values in C2 vertebra level (*P* = 0.156 to 0.985), or the FA values in the most severe cervical canal stenosis level (*P* = 0.129 to 0.780) (Table A in S1 File).

**Fig 4:**
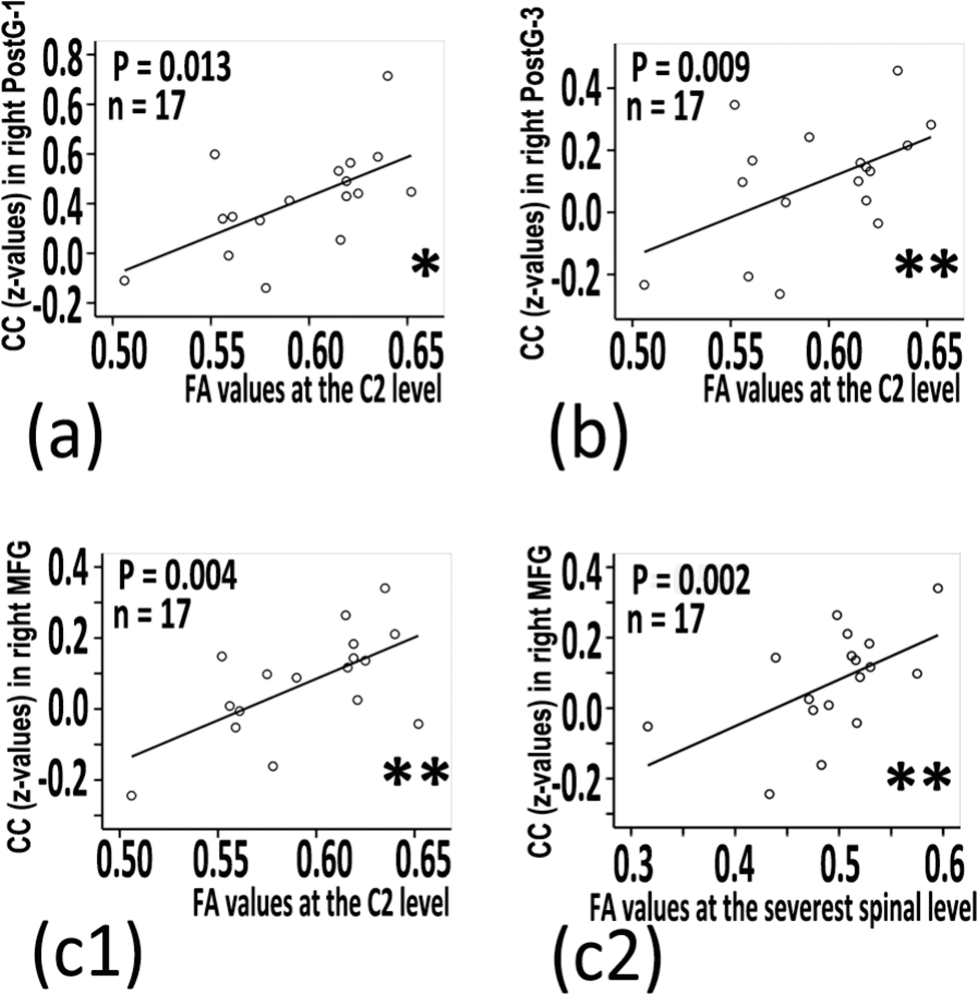
CSM-related thalamocortical connectivity coefficients related with clinical measures. Positive correlation between the fractional anisotropy (FA) value in the C2 vertebra level and CC z-values in right PostG1 (a),and right PostG3 (b) and right MFG (c1); Positive correlation between the FA value in the most severe cervical canal stenosis level and CC z-values in right middle frontal gyrus (MFG) (c2). (* P < 0.05, ** P < 0.01, with post-hoc correlation).

In the slow-4 band, there were significant positive correlations between the rsFC coefficients of the right middle frontal gyrus and the FA values at the C2 vertebra level (P = 0.004; β = 0.740, 95% confidence interval [CI]: 0.402 to 0.900), and FA values at the most severe cervical canal stenosis level (P = 0.002; β = 0.742, 95% confidence interval [CI]: 0.405 to 0.901). There were significant positive correlations between the rsFC coefficients of the right postcentral gyrus-1 (P = 0.013; β = 0.716, 95% confidence interval [CI]: 0.358 to 0.890), right postcentral gyrus-3(P = 0.009; β = 0.717, 95% confidence interval [CI]: 0.360 to 0.890) and FA values at C2 vertebra level. There were no significant correlations between the rsFC coefficients in the slow-4 band and the JOA score (*P* = 0.104 to 0.751, without correlation) or NDI score (*P* = 0.052 to 0.874, without correlation) (with post-hoc correction in Fig 4 and without correlation in Table B in S1 File).

## Discussion

With the discovery of thalamocortical connectivity disturbances in two slow frequency bands, this study provides a new insight into the neural pathophysiology of CSM as follows: (1) decreased rsFC in the slow-5 band, but increased rsFC in the slow-4 band, of thalamocortical circuits mainly in the sensorimotor and temporal cortex; (2) increased thalamo-prefrontal connectivity in both the slow-5 and slow-4 bands, but a larger volume and wider distributed in the slow-4 band; (3) increased connectivity in slow-4 band was related to the shorter disease duration and higher FA values in the cervical spinal cord, with hyperconnectivity of the slow-4 band in the thalamocortical system associated with earlier stage disease and mild spinal cord structural injury. These findings of thalamocortical disturbances in CSM are consistent with and expand upon previous reports of sensorimotor neuronal damage [6] and cortical reorganization [3,17,18].

### Discriminative connectivity information in sensorimotor and temporal circuits: revealed by two slow frequency bands

We found a significant decrease in thalamo-sensorimotor connectivity in the slow-5 band, a relatively lower frequency band, which may imply neuronal degeneration or axon/synaptic impairment in the circuit [9,13]. These findings are consistent and complementary with significantly lower activation within the postcentral gyrus [3] and a decrease in the N-acetylaspartate/creatine ratio in the motor cortex, reflecting neuronal damage [6]. However, it remains controversial whether thalamic and/or cortical neuronal damage is secondary to degenerative incomplete or compressive spinal cord injury. By contrast, in theslow-4band, we observed increased thalamocortical connectivity in the sensorimotor circuit. Hyperconnectivity of the sensorimotor circuit may imply cortical recruitment and regional reorganization in patients with CSM [2,3,18]. Further support for functional reorganization of sensorimotor region was reported in spinal cord injury[19–21]. An alternative explanation for the hyper-connectivity may also relate to reduce local inhibitory function during the inter-neuronal damage [22].

Several previous studies have examined the rs-fMRI dataset by decomposing the BOLD signal into different frequency bands[13,15,23] and have demonstrated that the power to analyze brain functional properties varies between different frequency bands[13,23,24]. Interregional rsFC strengths at slow-4 were demonstrated to be generally weaker than those at slow-5 in physiology conditions of spontaneous low-frequency BOLD fluctuations, and the sensorimotor network and the limbic system, which have most sensitive to spectrum effect on interregional functional connectivity [13]. The distinct frequency characteristics of functional connectivity in different brain areas might were influenced by the cytoarchitecture and synaptic linkage among them [25]. In the present study, the rsFC in the thalami-sensorimotor circuits was found decrease in slow-5, and yet increase in slow-4, suggesting that the discriminative connectivity disturbances of thalamo-sensorimotor circuits in two classic slow frequency bands in the patients with CSM.

In our study, the pattern of connectivity disturbances in the temporal circuits was similar to that in the sensorimotor circuits. The superior temporal gyrus have been linked to information processing ability, while the middle temporal pole and parahippocampa gyrus provide the major polysensory input to the hippocampus through their entorhinal connections, and are the recipients of differing combinations of sensory information [26]. Decreased rsFC in the right middle temporal pole and parahippocampa gyrus may imply functional cortical damage or insufficient sensory information input in patients with CSM. Increased rsFC in the contralateral temporal gyrus may imply cortical recruitment or compensation, which is involved the senior sensory and emotion information, including anxiety and depression processing, in cortical areas [1,27].

### Hyperconnectivity in prefrontal circuits was more prevalent in the slow-4 band

In fMRI experiments, the superior frontal gyrus is involved in self-awareness, which is coordination with the action of the sensory system [28], while the middle frontal gyrus is involved in integration executive mechanisms and operative and input information processing [29]. Increased rsFC in prefrontal circuits may imply cortical recruitment or reorganization in higher-order information processing. We found evidence of prefrontal connectivity with larger volume and wider distributed was demonstrated in slow-4 band compared to slow-5, suggesting that slow-4 analysis may provide more information as selected features to reflect the pathogenesis of CSM brain function.

### Increased connectivity is correlated with clinical index in the slow-4 band

The correlations between the increased connectivity in slow-4 and clinical measures suggest the potential for using thalamocortical functional connectivity as an underlying biomarker for predicting disease severity and disease progression, although more study is need to confirmed it. Especially when the rsFC in the thalamo-sensory circuit (involve the right postcentral gyrus)and thalamo-PMC circuit (involve the right middle frontal gyrus) were positively related to FA values in the cervical spinal cord, suggesting more enhanced connectivity occur in the minor spinal cord structural injury. Recently, spinal cord injury [30] and CSM [3,4] have been shown to induce changes in cortical activation during sensorimotor tasks. Freund [30] have also demonstrated that the extent of microstructural changes may reflect the plasticity of motor pathways associated with cortical reorganization in cervical cord injury. After CSM surgery, plasticity of central nervous system (CNS) might contribute to functional restoring, and it has been duly noted [3,17,31].

### Study limitations

It is worth mentioning that thalamocortical connectivity also was calculated only in routine low frequencies band (0.01–0.1Hz or 0.01–0.073),and without significantly between group differences was observed in CSM and controls (Data A in S2 File). It also needs to be noted that the segmented thalamic mask was developed from the FSL template based on anatomy connectivity [32]. Nevertheless, the contrast of thalamo-cortical connectivity between the subject groups showed consistency with what many other neuroimaging studies reported. Moreover, different thalamo-cortical functional connections provided consistent interpretations to neural activation patterns were observed in prior tasks studies [2,3,20]. Furthermore, this study was not classified by single- or multi-level myelopathy, unilateral or bilateral compressions due to the relatively small sample size, also thalamic mask is bilateral masks, which ignore the lateralized correlation between the brain change and cervical cord injury. Further whole brain analysis and postoperative decompression analyses are required in larger sample size and postoperative follow-up studies.

### Conclusions

In summary, this study explored the distributed alteration of thalamocortical connectivity in two difference bands, increase in slow-4 and decrease in slow-5, and the clinical relevance implying the thalamocortical functional reorganization during spinal cord degeneration inpatients with CSM. These results also suggest that thalamo-cortical disturbances revealed by two slow frequency bands have the prospect of becoming a potential MRI marker for therapeutic treatment effects of CSM in future clinical studies as well as SCI.

## Materials and Methods

### Participants

This study was approved by the institutional review board (First Affiliated Hospital, Nanchang University, China). Informed consent was obtained from all subjects. Seventeen right-handed consecutive patients with degenerative CSM (8 females, 9 males; age at 50.53±7.27 years) were recruited. The mean duration of symptoms from disease onset to the date of MRI examination was 9.06±9.86 months. The clinical severity of myelopathy patients was evaluated by JOA score system [33] (11.82±2.81(mean ± standard deviation)) and NDI questionnaires (32.6% ± 11.9% (mean ± standard deviation)). The JOA system evaluates the severity of myelopathy by assigning scores based on degree of dysfunction, and NDI was designed to measure activities of daily living in patients with neck pain. Inclusion criteria of patients were clear evidence of cord compression on a cervical spine MRI, including cervical spondylosis, or an ossified posterior longitudinal ligament. Two radiologists determined spinal cord compression when the cord surface was clearly indented or the cord diameter was narrowed by compression. Exclusion criteria were trauma- or infection-related cord compression or other neurological disorder such as multiple sclerosis, or a history of trauma.

Seventeen right-handed age-, and sex-matched control subjects with no previous clinical history of CSM or neurological disease were recruited (S1).

### Image acquisition

MRI scans were performed with a 3.0 Tesla MRI scanner (Trio Tim; Siemens, Erlangen, Germany). Subjects were instructed to keep their eyes closed, not to think about anything in particular, and not to fall asleep. A 240 time points rs-fMRI brain images were acquired using a standard T_2_*-weighted gradient echo sequence with the following parameters: repetition time/echo time = 2000/30 ms, field of view = 200 × 200 mm, matrix = 64 × 64, and 30 interleaved axial slices with 4-mm thickness with an interslice gap of 1.2-mm. Sagittal and axial conventional T_1_W, T_2_W and T_2_-FLAIR images were acquired in the brain and cervical spinal cord for diagnosis in each subject. Additional DTI images using a spin echo single-shot echo planar sequence were acquired to evaluate the cervical structural damage [34] (TR/TE = 5000/106 ms; NEX = 2; matrix = 128 × 124; FOV = 128 × 124 mm; slices = 16; slice thickness = 5 mm; orientation = axial; 20 nonlinear diffusion weighting gradient directions with *b* = 600 s/mm^2^ and 1 additional image without diffusion weighting [i.e., b = 0 s/mm^2^]). The image slice planning was the same as the anatomical axial T_1_W and T_2_W images, covering the cervical spinal cord from C1 to C7.

### Data preprocessing

The first 10 time points were discarded to allow the MR signal to reach steady state and participants to get used to the scanner noise. Rs-fMRI images were slice timing corrected, and motion correction was performed to adjust the time series of images using the Data Processing Assistant for Resting-State fMRI Advanced Edition (DPARSFA) V2.2 (http://www.restfmri.net)[34] running in Matlab 7.14.0 (Math Works, Natick, MA, USA). The images were then registered with the high-resolution T_1_ image using SPM8 (http://www.fil.ion.ucl.ac.uk/spm8/). Spatial smoothing was performed using a 6-mm full-width-half-maximum Gaussian kernel and temporal band-pass filtering (0.01 < f < 0.073 Hz) to reduce the effects of low-frequency drift and physiological high frequency noise [35].

The physiological routine low frequency oscillations (0.01–0.1 Hz) reflect spontaneous neuronal activity were subdivided by band-pass filtering into a relatively lower frequency slow-5 (0.01–0.027 Hz) band and a slightly higher frequency slow-4 (0.027–0.073 Hz) band for seed-based functional connectivity analysis [10,15].

### Thalamic seed-based functional connectivity analysis

We computed group level thalamo-cortical functional connectivity for both bands (slow-5 and slow-4) in the same manner as for the primary broadband functional connectivity analysis [35]. The thalamic seed (Data B and C in S2 File) was placed over the entire bilateral thalamus, within seven segments of the thalamus (seven subfields from the FSL template [32] demonstrated in Fig 1F), *Pearson's* correlation was computed between the preprocessed average time series of the seed and each voxel within seven exclusive cortical regions. The correlation coefficients values were z-transformed with Fisher's r-to-z transformation and were used for subsequent group-level analysis.

Seven exclusive cortical regions (Data D in S2 File); primary motor (M1, Fig 2-i), primary and secondary somatosensory (S1/S2, Fig 2-ii), occipital cortices, prefrontal (PFC, Fig 2-iii), premotor (lateral and medial) (PMC, Fig 2-iv), posterior parietal (PPC), and temporal (Fig 2-v)) were manually outlined on MNI standard *T*_1_-weighted images using anatomical landmarks, as detailed previously [32,36].

### Fractional anisotropy (FA) in the cervical spinal cord

FA metrics were calculated in DTI native space for each subject using TrackVis (http://www.trackvis.org/). Regions of interest (ROIs) were placed at axial nonstenotic levels of the entire spinal cord, typically in the C2 vertebra level and the level of most severe cervical canal stenosis (S2 File).

### Statistics analysis

Two-sample t-test was performed to statistically compare group-level motion correction, including six motion parameters involving three angular rotations (roll, pitch, and yaw in units of degrees) and three directional displacement (in millimeters) and dynamic frame displacement (summary of all six parameters in millimeters). For comparison of thalamocortical connectivity during resting state, two-sample *t*-test was used to compare rsFC maps between patients and controls. Every statistical significance was determined with Monte Carlo simulation [37] (AlphaSim corrected, single voxel *P* = .05, FWHM = 6 mm, 10,000 simulations, using every exclusive cortical region) combined with different cluster size (calculated by AlphaSim program, ⅰ≥ 20 voxels inM1 region, ⅱ≥ 16 voxels in S1/S2 region,ⅲ≥ 90 voxels in occipital region, ⅳ≥ 90 voxels in prefrontal region,ⅴ≥ 50 voxels in premotor region, ⅵ≥ 45 voxels in posterior parietal region, ⅶ≥ 80 voxels in temporal region, respectively; detailed AlphaSim information in Data E in S2 File), this correction was conducted using the AlphaSim program embedded into the REST package (http://restfmri.net).

Linear regression was performed to determine separate correlations between the thalamo-cortical connectivity coefficients (z-values) in the slow-5 or slow-4 band and clinical measures, including disease duration, JOA score, NDI score and the mean FA values in the cervical cord (*P*< 0.05 with post-hoc dropping outliers correlation [38], SPSS v13.0; SPSS Inc, Chicago, IL,USA).

## Supporting Information

S1 File Thalamic seed-based functional connectivity within connecting the exclusive cortical regions in routine low frequencies band. (PDF)

S2 File Data A, ClinicalData.xlsx: Clinical assessment of symptom period, FA values, NDI Scores, JOA scores. Data B, Thalamicseed, including 7 thalamic seed in 3-mm voxels. Data C, Thalamicseed-Cortex Mask, including 7 thalamic seed and Cortex Mask in 1-mm voxels. Data D, CortexMask, including 7 cortex mask in 3-mm voxels. Data E, AlphaSim, with calculation in different cortex mask.
(ZIP)
