# Supplementary material for: Characterizing Thalamocortical Disturbances in Cervical Spondylotic Myelopathy: Revealed by Functional Connectivity under Two Slow Frequency Bands
Source: PLoS One. 2015 Jun 8;10(6):e0125913. doi: 10.1371/journal.pone.0125913 (PMC4460123; doi:10.1371/journal.pone.0125913)
Supplement: S1 File — (PDF) [file pone.0125913.s001.pdf]

**Thalamic seed-based functional connectivity within connecting the exclusive cortical regions in routine low frequencies band**

We computed group level thalamo-cortical functional connectivity in routine low frequencies band (0.01–0.10 Hz or 0.01-0.073 Hz) in the same manner as for the slow-4 or slow-5 bands functional connectivity analysis. The thalamic seed was placed over the entire bilateral thalamus, within seven segments of the thalamus (seven subfields from the FSL template[1] demonstrated in Figure 2f), Pearson's correlation was computed between the preprocessed average time series of the seed and each voxel within seven exclusive cortical regions. The correlation coefficients values were z-transformed with Fisher's r-to-z transformation and were used for subsequent group-level analysis.

Seven exclusive cortical regions (primary motor (M1, Figure 3-i), primary and secondary somatosensory (S1/S2, Figure 3-ii), occipital cortices, prefrontal (PFC, Figure 3-iii), premotor (lateral and medial) (PMC, Figure 3-iv), posterior parietal (PPC), and temporal (Figure 3-v)) were manually outlined on MNI standard $T_1$-weighted images using anatomical landmarks, as detailed previously.[1, 2]

**Statistics analysis**

For comparison of thalamic seed-based functional connectivity with the exclusive cortex during resting state, two-sample t-test was used to compare functional connectivity coefficients maps between patients and controls ($P < .05$, AlphaSim corrected). Further, the significantly altered brain areas in the exclusive cortex were used to select the masks to be retained for two-sample t-test compare analysis.

**Results**

Compared with healthy controls, no region with altered functional connectivity (both in 0.01–0.10 Hz or 0.01-0.073 Hz) was found between the all the thalamic segments and it’s exclusive cortices in the cervical myelopathy group. The differences in t-values and *P*-values of the patient vs healthy controls connectivity coefficients in the low frequencies band (for example 0.01–0.10 Hz) band are listed in Table C.

*Table A. Clinical associations of thalamocortical connectivity coefficients (z-values) in slow-5 frequency-band*

| | Duration of symptoms (month) | | JOA score | | NDI score | | FA values in C2 level | | FA values in the most severe level | |
|---|---|---|---|---|---|---|---|---|---|---|
| | *r* | *P* | *r* | *P* | *r* | *P* | *r* | *P* | *r* | *P* |
| *Right precentral gyrus* | -0.370 | 0.144 | 0.226 | 0.383 | -0.334 | 0.190 | -0.073 | 0.780 | -0.299 | 0.244 |
| *Left precentral gyrus* | -0.216 | 0.405 | -0.009 | 0.974 | 0.100 | 0.703 | 0.275 | 0.286 | -0.190 | 0.466 |
| *Left postcentral gyrus* | -0.051 | 0.845 | -0.231 | 0.372 | 0.006 | 0.981 | -0.248 | 0.337 | -0.138 | 0.598 |
| *Right postcentral gyrus* | -0.327 | 0.200 | -0.240 | 0.353 | 0.254 | 0.325 | 0.383 | 0.129 | -0.360 | 0.158 |
| *Right middle frontal gyrus* | 0.323 | 0.206 | -0.093 | 0.722 | 0.280 | 0.276 | -0.176 | 0.500 | 0.264 | 0.306 |
| *Right inferior frontal gyrus* | 0.040 | 0.880 | 0.161 | 0.538 | -0.057 | 0.829 | 0.307 | 0.230 | -0.005 | 0.985 |
| *Left superior frontal gyrus/ supplementary motor area* | -0.089 | 0.735 | 0.021 | 0.935 | -0.249 | 0.335 | 0.288 | 0.262 | -0.061 | 0.815 |
| *Right middle temporal pole* | -0.029 | 0.911 | -0.291 | 0.247 | 0.133 | 0.610 | -0.160 | 0.540 | -0.227 | 0.382 |
| *Right parahippocampa gyrus* | -0.206 | 0.428 | 0.163 | 0.533 | -0.161 | 0.537 | 0.154 | 0.554 | -0.194 | 0.456 |

*Notes: FA = Fractional Anisotropy; JOA = Japanese Orthopaedic Association; NDI = Neck Disability Index*

*Table B. Clinical associations of thalamocortical connectivity coefficients (z-values) in slow-4 frequency-band*

| | Duration of symptoms (month) | | JOA score | | NDI score | | FA values in C2 level | | FA values in the most severe level | |
|---|---|---|---|---|---|---|---|---|---|---|
| | *r* | *P* | *r* | *P* | *r* | *P* | *r* | *P* | *r* | *P* |
| *Right precentral gyrus* | -0.341 | 0.172 | 0.115 | 0.659 | -0.041 | 0.874 | 0.295 | 0.251 | 0.071 | 0.788 |
| *Right postcentral gyrus-1* | -0.143 | 0.584 | 0.128 | 0.623 | 0.061 | 0.851 | 0.607 | 0.010 (0.013) * | 0.470 | 0.057 |
| *Right postcentral gyrus-2* | -0.464 | 0.061 | -0.139 | 0.594 | 0.171 | 0.511 | 0.410 | 0.102 | 0.181 | 0.487 |
| *Left postcentral gyrus* | -0.533 | 0.028 (0.132) * | -0.192 | 0.461 | 0.091 | 0.727 | -0.008 | 0.976 | 0.209 | 0.420 |
| *Right postcentral gyrus-3* | -0.440 | 0.077 | 0.099 | 0.705 | -0.064 | 0.808 | 0.504 | 0.039 (0.009) * | 0.502 | 0.040 (0.232) * |
| *Right superior/ middle frontal gyrus* | -0.196 | 0.452 | -0.205 | 0.429 | 0.381 | 0.131 | 0.061 | 0.815 | 0.332 | 0.192 |
| *Left superior frontal gyrus* | 0.057 | 0.827 | 0.193 | 0.458 | -0.185 | 0.478 | -0.325 | 0.203 | 0.021 | 0.935 |
| *Right superior frontal gyrus* | 0.016 | 0.952 | -0.408 | 0.104 | 0.461 | 0.064 | -0.211 | 0.415 | 0.109 | 0.676 |
| *Right middle frontal gyrus* | 0.163 | 0.533 | -0.082 | 0.754 | 0.282 | 0.273 | 0.620 | 0.008 (0.004) * | 0.550 | 0.022 (0.002) * |
| *Right superior frontal gyrus* | -0.040 | 0.878 | -0.261 | 0.312 | 0.216 | 0.406 | 0.214 | 0.409 | 0.204 | 0.431 |
| *Left superior temporal gyrus* | -0.249 | 0.335 | -0.089 | 0.1734 | 0.062 | 0.813 | -0.183 | 0.482 | -0.481 | 0.051 |

*Notes: FA = Fractional Anisotropy; JOA = Japanese Orthopaedic Association; NDI = Neck Disability Index; * = with post-hoc correction*

*Table C The cervical myelopathy patients compared with the controls in low frequencies band (0.01–0.10 Hz), the altered functional connectivity in slow-4 and slow-5 bands as mask*

| | The mask form slow-4 altered cortex areas | t-value | p-value | The mask form slow-5 altered cortex areas | t-value | p-value |
|---|---|---|---|---|---|---|
| Primary motor cortex | Right Precentral Gyrus | 1.227 | 0.229 | Left Precentral Gyrus | 0.497 | 0.623 |
| | | | | Right Precentral Gyrus | 0.196 | 0.846 |
| Primary and secondary somatosensory | Right Postcentral Gyrus-1 | 1.485 | 0.147 | Left Postcentral Gyrus | -0.751 | 0.458 |
| | Right Postcentral Gyrus-2 | 1.360 | 0.183 | Right Postcentral Gyrus | -1.649 | 0.109 |
| | Right Postcentral Gyrus-3 | 1.718 | 0.095 | | | |
| | Left Postcentral Gyrus | 1.495 | 0.145 | | | |
| Prefrontal | Right Superior/ Middle Frontal Gyrus | 1.344 | 0.188 | Right Middle Frontal Gyrus | 1.247 | 0.221 |
| | Right Superior Frontal Gyrus | 1.352 | 0.186 | | | |
| | Left Superior Frontal Gyrus | 1.993 | 0.055 | | | |
| Premotor | Right Superior Frontal Gyrus | 0.958 | 0.345 | Right Inferior Frontal Gyrus | -1.954 | 0.059 |
| | Right Middle Frontal Gyrus | 1.687 | 0.101 | Left Superior Frontal Gyrus/ Supplementary Motor Area | -1.847 | 0.074 |
| Temporal | Left Superior Temporal Gyrus | 1.246 | 0.222 | Right Middle Temporal Pole | -1.532 | 0.135 |
| | | | | Right Parahippocampa Gyrus | -1.847 | 0.074 |

*Notes：BA = Brodmann area; MNI = Montreal neurological institute*

## Reference


1. Behrens T, Johansen-Berg H, Woolrich M, et al. Non-invasive mapping of connections between human thalamus and cortex using diffusion imaging. Nat Neurosci. 2003;6: 750-757.
2. Johansen-Berg H, Behrens TE, Sillery E, et al. Functional-anatomical validation and individual variation of diffusion tractography-based segmentation of the human thalamus. Cereb Cortex. 2005;15: 31-39.
